# Sexual and Psychosocial Risk Burdens Associated With Online Sex Seeking Among Young Men Who Have Sex With Men: Cross-Sectional Study

**DOI:** 10.2196/59072

**Published:** 2025-08-20

**Authors:** Doug H Cheung, Worawalan Waratworawan, Michael C Clatts, Donn Colby, Giang Minh Le, Yamol Kongjareon, Lan Anh Thi Do, Thomas E Guadamuz

**Affiliations:** 1 Mahidol Center for Health, Behavior and Society, Faculty of Tropical Medicine Mahidol University Bangkok Thailand; 2 JC School of Public Health and Primary Care Chinese University of Hong Kong Hong Kong China (Hong Kong); 3 Center for Applied Research on Men and Community Health Ho Chi Minh City Vietnam; 4 Center for Training and Research on Substance Abuse and HIV Hanoi Medical University Hanoi Vietnam; 5 Faculty of Public Health Pham Ngoc Thach University of Medicine Ho Chi Minh City Vietnam

**Keywords:** young men who have sex with men, Vietnam, Ho Chi Minh City, HIV, sexually transmitted infection, STI, mental health, gay dating app, geosocial networking app, preexposure prophylaxis, latent class analysis

## Abstract

**Background:**

The heightened HIV vulnerability associated with men who have sex with men (MSM) who find sex online in Western and East Asian countries may pose similar concerns for MSM in Southeast Asia. However, this line of research is underexamined among Southeast Asian MSM, especially in Southeast Asian cities with a high HIV prevalence among young MSM, such as Ho Chi Minh City, Vietnam.

**Objective:**

This study aimed to characterize the sexual behavioral and psychosocial correlates of online sex seeking in a sample of largely gay-identified young MSM in Ho Chi Minh City, Vietnam, and examine the relationships among social support, outness, and last instance of condomless anal sex while not on preexposure prophylaxis (PrEP) for HIV or antiretroviral therapy (ART). The analysis included specific attention paid to the use of different types of online sex platforms, which may contribute to the understanding of online sex seeking.

**Methods:**

Patterns of online sex seeking in a cross-sectional sample of young MSM (N=1005) were identified using latent class analysis. Multinomial logistic regressions and Poisson regressions with robust variance were used to estimate the associations between patterns of online sex seeking and other participant characteristics.

**Results:**

We found four latent classes of MSM with distinct profiles of online platform use for sex seeking: (1) *negligible app users*, (2) *gay app users*, (3) *poly app users*, and (4) *low-cost app users*. Patterns of online sex seeking moderated the protective effects of social support and outness on last instance of condomless anal sex while not on PrEP or ART. When stratified by level of social support and outness, only gay app users were associated with a protective effect on last instance of condomless anal sex while not on PrEP or ART when social support and outness were high, respectively. Low-cost app users were marginally associated with a higher prevalence of condomless anal sex while not on PrEP or ART when the level of outness was low.

**Conclusions:**

Young Vietnamese MSM who find sex partners online have distinct patterns of app use, with contrasting sexual and psychosocial health burdens that indicate that online sex seeking is a socially patterned behavior. HIV prevention programs and tailored digital interventions should consider the different exposures to social influences associated with patterns of social networking app use as they could moderate the effectiveness of the delivered programs and interventions for reducing HIV vulnerability in young MSM.

## Introduction

### Background

Worsening epidemics of HIV and sexually transmitted infections (STIs) and the growing use of recreational substances before or during sex (sexualized illicit drug use) among men who have sex with men (MSM) in Asia could converge through the use of social networking apps in general as well as gay-oriented geolocation-based social networking apps (gay apps). However, these connections remain underexamined in Southeast Asia, which may play a pivotal role in the prevention of HIV or STI transmission and drug-related harm [1-3], especially in highly populated urban centers with high concentrations of MSM and HIV prevalence among MSM populations, such as Ho Chi Minh City, where HIV prevalence among MSM was estimated to be 14.5% in 2009 and remains high [4,5]. Particularly noteworthy is that HIV prevalence among young MSM (aged 15-24 years) in Vietnam, a demographic group that relies heavily on app-based partner selection, quadrupled from 3% in 2011 to 13% in 2020 [6] concurrently with the escalating popularity of the use of social networking apps to find sex partners, with one Hanoi-based study showing a prevalence of 35% in 2008 rising to 76.6% in 2016 [7]. Social networking apps for sex seeking are of particular relevance to young MSM because these apps could be the primary resources to connect with their peers and could be an essential tool for some young MSM to explore and express their same-sex attraction. This is particularly important in Southeast Asia, where lesbian, gay, bisexual, transgender, and queer–supportive physical establishments are few, segregated, and stigmatized. However, young MSM’s engagement with social networking apps to find sexual partners could also expose them to sexual networks with a high risk of transmission of diseases such as HIV, STIs, and mpox.

Gay apps have been postulated to be a virtual risk environment for HIV and STIs; they are often analyzed as a binary exposure variable for HIV vulnerability [8]. At the same time, there is consistent evidence that MSM who use gay apps (vs nonusers) more frequently report a wide range of sexual behaviors that increase MSM’s HIV vulnerability: multiple sexual partnerships [9-13], group sex [14,15], recreational drugs before or during sex (“chemsex” or sexualized drug use) [11,14,16-18], a previous STI diagnosis [12,16,19], and being unaware of their sex partner’s HIV status [14].

There is contrasting evidence on the difference between gay app users and nonusers in the reported rates of condomless anal sex, a high-risk sexual behavior for HIV transmission. While some studies have found that condomless anal sex is higher among gay app users than among nonusers [9,15,19,20], others have found either no difference [10] or a lower rate among gay app users than among nonusers or those who find sex partners offline [11,21]. Gay app users also more frequently adopt HIV-preventive measures than nonusers, including HIV testing [10,11,19], HIV preexposure prophylaxis (PrEP) uptake [12,22,23], and seroadaptive behaviors such as serosorting [23]. Most gay apps allow users to display their HIV, PrEP, and viral load status; last HIV testing date; and safe sex or sexual risk preferences—informing others so that they can discuss safe sex or adopt seroadaptive strategies, such as having condomless anal sex only when both individuals use PrEP or have an undetectable viral load (also known as biomed matching) [24], which may facilitate higher rates of condomless anal sex but is considered a low-probability sexual behavior for seroconversion [22,23]. More importantly, most studies on gay apps have been conducted before larger-scale PrEP implementation or in areas with low PrEP coverage; therefore, they do not consider the dynamics of condomless anal sex with respect to inadequate PrEP uptake, which is a more relevant high-probability sexual behavior for HIV seroconversion in regions with increasing PrEP coverage such as Vietnam [9,15,19,20].

Affiliation with sex venues is known to converge sexual networks (increased density) to increase the probability of HIV seroconversion among MSM who engage in condomless anal sex [25]. Social networking apps serve a function similar to that of physical sex venues in connecting users and their sexual networks. Studies of phylogenetic linkages and venue-network analyses have found that internet venues (vs sauna) and specific apps used by MSM to find sex partners increase MSM’s risk of HIV seroconversion or STI transmission more than physical venues or other apps through closer proximity to sexual networks with active HIV or STI transmissions [26-28]. Among STI clinic–recruited MSM in the United States, reporting the use of Scruff and Grindr (gay apps) was found to be indicative of testing positive for STIs and having the users with the most significant degrees of connectivity and proximity (transmission probability) within a sexual affiliation network but not among MSM who had lower degrees of connectivity and proximity through the use of other apps [27]. On the other hand, social influences, such as how one’s behavior is affected by others (eg, condom use), could propagate seroconversions among MSM between sexual networks connected via social networking apps by increasing MSM’s HIV vulnerability or tendency toward sexual risk taking. Among MSM recruited from Grindr in the United States, those who included an app-met partner in their close network were nearly 5 times more likely to have condomless anal sex with their last app-met partner, indicating a strong social influence from app-met partners [29]. MSM can also be directly exposed to social influence through interactions with others on social networking apps, such as invitations to a drug or sex party, or through exposure to normative contexts (eg, profiles and online content-condom use) within app users [18].

As much as interpersonal relations may negatively influence one’s behavior, they could also be protective. Social support is often used as a measure of how a social network or group exerts its influence on health behaviors [25]. A prospective BROTHERS study involving 1000 Black MSM from the United States found that those who received social support from a higher number of social network members had a reduced risk of HIV seroconversion during the 1-year follow-up period [30]. Young MSM can also receive social support and other benefits from a social network directly from their engagement with social networking apps while seeking sex. Gerke et al [31] found that the increased frequency of social networking app use for sex seeking was associated with increased levels of social support among MSM from the United States.

In addition to being a social network, social networking apps can be conceptualized as social environments for young MSM to access and interact and connect with communities sharing common interests. Social networks, including gay apps, have also been postulated to play a role in young MSM’s identity development, such as facilitating “outness,” the degree of disclosing one’s sexual orientation to others [32,33]. Chan [32] found that outness moderated the relationship between the intensity of app use and the number of casual sex partners, showing that MSM who were less out had a more intense engagement pattern in app use, resulting in subsequent sexual behaviors. Sexual orientation concealment, the opposite measure of outness, has been shown to mediate between country-level stigma and sexual risk taking [34]. While earlier studies have shown that MSM who are less out use higher-risk physical venues to meet sex partners, it is not known whether young MSM who are less engaged in high-risk patterns of online sex seeking may be predisposed to HIV infection [35]. Similarly, young MSM with lower levels of social support could be more vulnerable to social influence online than those with higher levels of social support. However, previous research on online sex seeking has primarily focused on *whether* a heightened HIV risk exists compared to non–app users; scant studies have examined the underlying social processes, such as *how* (eg, different exposures to online social contexts) online sex seeking could increase HIV vulnerability among MSM and for *whom* (eg, young MSM who are less out) [36-38].

### Objectives

Social networking apps are explicitly designed as social structures (social networks and communities) to encourage user interaction [39]. Therefore, an app’s social structural characteristics are robust determinants of individuals’ use, which digital marketers have exploited to target consumer behaviors [25,40-42]. Therefore, young MSM app use patterns could underlie continued exposure to the social environments of these apps, consisting of a collection of sexual networks. In this study, we used latent class analysis (LCA) to identify hidden subgroups of young MSM from Ho Chi Minh City, Vietnam, when grouping young MSM who share a similar pattern of online sex seeking. LCA is an empirical, nonparametric statistical procedure used to classify participants into subgroups based on their responses to questions. It indicates a hypothesized social structure, identity, or shared exposure or risk level that is mutually exclusive, for example, polygamous versus monogamous daters [43]. LCA has been used to identify the social network influence on sexual and drug use behaviors [44], categories of normative network influence [45], and patterns of online and offline connectedness among MSM [37]. We hypothesized that the patterns of online sex seeking identified through LCA have distinctive sociodemographic, sexual behavioral, and psychosocial characteristics. The most distinct characteristics would include sexual network characteristics (sexual partnership type and size) and network-relevant psychosocial characteristics (sexualized drug use and alcohol consumption), as predicted by principles of social network theories such as homophily (clustering by similar behaviors) [17,46]. We further hypothesized that the identified app use patterns would affect the relationships among social support, outness, and condom use behavior (last instance of condomless anal sex while not on PrEP or antiretroviral therapy [ART]) through a moderating relationship (limiting or amplifying) among young Vietnamese MSM. That is, the effects of social support and outness on condom use behavior depend on the specific category of app use patterns, and vice versa.

## Methods

### Study Setting and Participants

Data were collected from a cross-sectional survey of young MSM in Ho Chi Minh City, Vietnam, between March 2023 and June 2023. The eligibility criteria were (1) male sex at birth, (2) age of 16 to 29 years, (3) anal intercourse with a man in the previous 6 months, and (4) residence in Ho Chi Minh City for at least 6 months.

### Procedure

The study design, procedures, and survey questionnaire were informed by formative research involving 4 focus group discussions that included 2 groups of young MSM and 2 groups of health care providers. A community advisory board comprising workers from a nongovernmental organization (NGO) and youth leaders also contributed to the study’s development. Data were collected via a web-based surveys using Qualtrics (Qualtrics International Inc) [47].

Participants were primarily recruited through a local NGO, the Center for Applied Research on Men and Community Health. The NGO recruited participants in three ways: (1) online recruitment, (2) offline recruitment, and (3) affiliated network recruitment. Online recruitment consisted of posts on official websites, social media accounts, and chat groups on Line and WhatsApp and paid banner advertisements on Blued, Facebook, and Zalo. Offline recruitment consisted of outreach activities at public universities and cafés owned by the NGO. During the offline outreach, experienced and trained NGO staff provided potential participants with a QR code to scan from their smartphones to link to the online survey. Participants were also offered access to tablets to complete the online survey if, for any reason, they did not have a smartphone or did not want to (or could not) take the online survey from their own smartphones. Affiliated network recruitment involved sending a link to the online survey via Zalo and Facebook messengers from NGO-affiliated personnel to students at local public and private universities in Ho Chi Minh City, Vietnam. Prospective participants who clicked on the link or banner or scanned the QR code were led to a study information sheet that detailed the study’s risks, benefits, and contact information. Participants were promised anonymity and informed that their refusal to take part at any point would not result in any penalty or loss of opportunity to use services or for educational advancement.

### Sample Construction

Of a total of 2446 responses recorded from the Qualtrics survey web page links, 560 (22.9%) were incomplete responses, including 373 (66.6%) that failed to submit screening questions and 187 (33.4%) that were eligible but the survey responses still needed to be submitted before the survey timed out. Of the remaining 1886 responses, 803 (42.6%) were ineligible, and 20 (1.1%) refused to provide consent, and of those that completed the survey, 58 (3.1%) were found to have duplicate contact information and were excluded from the study. The inclusion of the 187 eligible incomplete responses resulted in a response rate of 79.1% (1005/1270) and a total sample of 1005 unique responses or participants.

### Ethical Considerations

This study was conducted in accordance with ethical principles for human participant research and received full ethics approval from the Mahidol University Faculty of Social Sciences and Humanities Institutional Review Board (Thailand; approval 2021/119.2010) and the Center for Creative Initiatives in Health and Population review board (Vietnam; approval 09032022). This research was classified as human participant research requiring full board review, with no exemptions granted. Both ethics committees granted a waiver of parental permission for participants aged 16 to 17 years, allowing these adolescents to consent independently. All participants provided electronic informed consent before taking part by selecting a checkbox on the online consent form, which detailed the study purpose, procedures, risks, and benefits and the voluntary nature of participation. The consent process explicitly stated that refusal to participate or withdrawal at any time would not result in any penalty or loss of access to services or educational opportunities. For secondary analyses of the collected data, the original informed consent and institutional review board approvals included provisions allowing for such analyses without requiring additional consent from participants. To ensure privacy and confidentiality, the survey was designed to be completely anonymous, with no personal identifiers collected during the main survey process, ensuring that the data could not be traced back to individual participants. All data were stored in encrypted, password-protected files accessible only to authorized research team members. This study did not collect or present any images of participants that could lead to identification. Participants were offered compensation in the form of a VND 50,000 (approximately US $2) online gift card upon completion of the survey, which took approximately 30 minutes. To maintain anonymity while providing compensation, participants had the option to provide contact information on a separate platform disconnected from their survey responses. No images identifying individual participants were included in any study materials or publications. Had such images been necessary, explicit written consent would have been obtained from identifiable individuals, and these consent forms would have been submitted with the manuscript.

### Measures

#### Sociodemographics

Participants were asked to self-report sociodemographic characteristics, including age (in years), employment status, educational level, monthly income, sexual orientation, and whether they had ever (in their lifetime) received or provided sex in exchange for goods or opportunities (eg, money, drugs and alcohol, mobile phones, mobile phone credits, clothes, and grades or educational opportunities).

#### Sexual Partnering and Practices

Participants reported the number of male sexual partners in the previous year (range 1 to 200). Partner numbers were categorized as a dichotomous variable consisting of having had between 1 and 10 male sex partners in the previous year or having had ≥11 male sex partners in the previous year. We asked the participants about the HIV status of their last male sexual partner (“HIV infected,” “uninfected,” or “don’t know or not sure”). Participants were asked whether they or their partners had used condoms during their last anal intercourse (“Yes, for the entire time we had anal sex,” “Yes, some of the time when we had anal sex,” or “No, we did not use a condom”). Those who answered with “Yes, some of the time when we had anal sex” or “No, we did not use condoms” were categorized as having had condomless anal sex in their last sexual encounter. Participants were asked whether they had ever participated in group sex activities (“many times (>10 times),” “sometimes (5-10 times),” “a few times (<5 times),” and “never”).

#### History of STIs

Participants were asked to report whether they had been tested and diagnosed with the following STIs in the previous 12 months: syphilis, gonorrhea, chlamydia, genital warts, perianal warts, genital herpes, hepatitis B, hepatitis C, and pubic lice or scabies. Participants who reported any of the aforementioned STIs were categorized as having had any STIs in the previous 12 months.

#### Behavioral and Biomedical HIV Prevention Practices

Participants were asked to select the date of their last HIV test; those who set their testing date within the previous 1 to 12 months were categorized as having been HIV tested within the previous 12 months. Participants who had ever been tested for HIV were asked to self-report their HIV status as “positive,” “negative,” or “unknown.” Participants who reported HIV positivity were subsequently asked whether they were currently on ART. For participants reporting an HIV-negative or unknown status, they were subsequently asked whether they were taking PrEP (“Yes, I am currently taking,” “Yes, I used to take PrEP but not now,” “No, I don’t take PrEP,” or “No, I don’t know about PrEP”). Those who reported “Yes, I am currently taking” were categorized as currently taking PrEP. We categorized those who reported condomless anal sex during their last sexual encounter but simultaneously reported not currently taking PrEP or being on ART as having condomless anal sex in their last sexual encounter without biomedical prevention strategies. Those who self-reported having HIV infection and were currently taking ART were assumed to have an undetectable viral load and were categorized to not currently taking PrEP or being on ART in having condomless anal sex in their last sexual encounter without biomedical prevention strategies.

#### Assessment of Mental Health, Stigma, and Behavioral Risk Factors

Suicidality was assessed using the 4-item Suicide Behaviors Questionnaire–Revised [48]. Each item assessed a separate suicidal behavior: (1) lifetime suicide ideation and suicide attempts, (2) frequency of suicidal ideation over the previous 12 months, (3) threats of suicide attempts, and (4) likelihood of suicide ideation in the future. A total score ranging from 3 to 18 was calculated. A cutoff score of ≥7 indicates high sensitivity and specificity for suicidality among nonclinical samples. We found adequate internal consistency for this scale within our sample (Cronbach α=0.82). Depression was assessed through past-7-day depression symptoms using the 10-item Center for Epidemiologic Studies Short Depression Scale [49]. The total score ranges from 0 to 30, with a score of ≥10 indicating elevated depressive symptoms. Perceived HIV stigma was assessed using the 7-item Public HIV Stigma Scale [50]. Participants were asked to rate whether they agreed with statements that described how an HIV-infected individual should be treated by their spouse, friends, family, and society, ranging from 0 to 3 (“strongly agree” to “strongly disagree”). The following is a sample item from this scale: “An HIV-infected person’s family would not care for them.” Total scores ranging from 0 to 21 were calculated. We found excellent internal consistency for this scale within our sample (Cronbach α=0.92). Hazardous alcohol use was measured using the 3-item Alcohol Use Disorders Identification Test–Consumption scale [51]. Participants were asked to report their frequencies of alcohol consumption and binge drinking and drinking amount during a typical drinking episode. The total score was calculated in the range of 0 to 12. A total score of ≥4 indicates hazardous or active alcohol use disorders [51]. Illicit sexualized drug engagement was assessed by asking whether the participants had ever used the following drugs: amphetamine, crystal meth—inhaling or smoking, crystal meth—injection, ecstasy or 3,4-methylenedioxymethamphetamine, ketamine, and γ-hydroxybutyric acid. Participants who reported having used any of the aforementioned drugs for sex were categorized as having lifetime illicit sexualized drug engagement.

#### Social Support and Outness

Social support was measured using the 19-item Medical Outcomes Study Social Support Survey [52]. Participants were asked to rate whether social support was available to them if needed on a scale from 1 to 5 (“none of the time” to “all of the time”); a final score was calculated by averaging the score of each of the 19 items. The following is a sample item from this scale: “Someone you can count on to you when you need to talk.” We found excellent internal consistency in this scale among our participants (Cronbach α=0.98). Outness was assessed using questions based on our team’s formative research on Southeast Asian MSM. The questionnaire consisted of 6 items and asked participants whether they had told or expressed to others that they were MSM, to what extent, and to whom. The 5 items asked the participants to rate to what extent they had expressed their identity to (1) other students at school or university; (2) teachers or staff at school or university; (3) people, bosses, or supervisors at workplaces; (4) friends outside school, university, or the workplace; and (5) people in their family. Participants could choose from a scale of 1 (“Yes, told or expressed it to everyone”) to 4 (“Did not tell or express it to anyone”). We found excellent internal consistency for this measure in our sample (Cronbach α=0.95).

#### Online Platforms Used for Sex Seeking

Our team’s previous formative research among young MSM in Ho Chi Minh City, Vietnam, provided a measurement of online platform use. Participants were asked which of the following apps they actively used to meet their sexual partners (participants were allowed to choose more than one option): Hornet, Blued, Jack’d, Grindr, Tinder, Twitter (subsequently rebranded X) groups, Facebook groups, Zalo groups, and Line groups.

### Data Analysis

#### The Identification of the Latent Class Model

LCA was used to describe patterns of online platform use for sex seeking using the *PoLCA* package in R (R Foundation for Statistical Computing), which uses expectation maximization and Newton-Raphson algorithms to find maximum likelihood estimates of the latent class model [53]. A 3-step approach to LCA was used to identify latent classes and examine their associations with a distal outcome, which is considered the current best standard in terms of having the most negligible bias and minimal other statistical disadvantages compared to other LCA approaches [54]. The LCA procedures were guided by the best practices recommended by Weller et al [43]. Model fit criteria and diagnostics of estimated models with 2 to 6 latent classes, each with 5 random starts and a maximum of 5000 iterations, are summarized in Table S1 in Multimedia Appendix 1. Bayesian information criterion (BIC) and conditional Akaike information criterion values were used to select the final latent class model; lower values indicate better model fit. An entropy cutoff value of 0.80 was used to confirm that the chosen latent class model adequately classified the participants in each latent class with high certainty. In addition, Vuong-Lo-Mendell-Rubin likelihood ratio tests were conducted to compare the statistical performance of the selected latent class model with those of other models [55].

#### The Characterization of Participants’ Assignment to a Latent Class Model

Multinomial logistic regression was used to characterize the associations between participant characteristics and latent class membership using the multinomial logit link function to estimate model parameters, including relative risk ratios, adjusted relative risk ratios, and the corresponding 95% CIs. To test the hypothesis that latent class assignments could be best predicted and explained by social network–relevant variables, 2 methods were used. First, a purposeful model selection approach was used to obtain the most parsimonious multivariable multinomial logistic regression model based on the predictive values of the predictor variables (significance of parameter estimates) [56]. Variables included in the final model were assessed by breaking down the model’s explained variance (*R*^2^) through relative importance weight analyses (*relaimpo* statistical package in R by Groemping [57]) in separate logistic regression models that coded each latent class as a dummy outcome variable [57,58]. Second, predictor variables were grouped by sets of theoretically related variables to identify the most distinct set of variables to explain the latent class assignment, as predicted by social network theories [25,39,59], such as the number of sexual partners (size) and types of partnerships (ties). A final model was constructed based on the following sets of variables: (1) age, (2) socioeconomic status, (3) sexual orientation and outness, (4) sexual behavior and sexual health histories, (5) uptake of HIV-preventive measures, (6) number of sexual partners and sexual partnership types, (7) substance use, (8) mental health, and (9) psychosocial statuses [25,39,59].

The models’ adjusted *R*^2^ and BIC values divided by the number of model parameters (df) were used to assess each model’s (each set of variables’) relative contribution in explaining (*R*^2^) and predicting (BIC) latent class assignment [60,61].

#### Moderation Analyses

The moderating relationships among social support, outness, patterns of online sex seeking, and last instance of condomless anal sex (denoted as “condomless anal sex”) while not on PrEP or ART were examined using Poisson regression with robust sandwich variance to estimate the prevalence ratio or incidence risk ratio, which provides more reliable estimates than logistic regression models when the outcome prevalence is of >10% [62]. Separate models were constructed and stratified by social support and outness (high and low levels defined by sample means) to evaluate the conditional association between latent class and last instance of condomless anal sex without PrEP or ART.

Confounders were identified through a literature review and conceptualized if they temporally preceded and were associated with the exposures, mediators, or outcomes but not on the downstream causal paths among exposure, mediator, and outcome. For example, the number of sexual partners and sexualized drug use are facilitated by app use and increase the probability of condomless anal sex; therefore, they were not conceptualized as confounders [63]. Multicollinearity was checked by computing the variance inflation factor; covariates with a variance inflation factor value of ≥10.0 were rescaled or removed if they were theoretically redundant [64]. We assessed the model fit of the final multivariable model using the Hosmer-Lemeshow goodness-of-fit test [65]. All analyses were conducted using the R statistical software (version 4.2.0) with 0.05 critical values for hypothesis testing.

#### Sensitivity Analyses

The sensitivity of the multivariable moderation analyses was evaluated using different cutoffs of social support and outness by 15% increments and decrements from each measure’s mean to determine the range of statistical significance. Sensitivity to sampling bias due to variations in recruitment methods (eg, offline recruitment at universities) was evaluated in 2 ways. First, moderation and mediation analyses were repeated on a restricted sample size to exclude participants who reported postgraduate education. Split sample analyses were conducted by equally distributing the probability of the outcome (last instance of condomless anal sex without PrEP or ART) into smaller samples (50%-90% of the total sample) with random draw replacement [66]. The same moderation analyses were conducted in these resampled smaller samples to determine whether the associations detected in the total sample were robust to sampling variations. The same moderation analyses were conducted with participants who reported using Blued, Jack’d, Grindr, and Hornet to determine whether the use of these gay apps would yield the same moderating effects as patterns of online sex seeking identified via LCA procedures.

## Results

### Participant Characteristics

Table 1 presents the characteristics of the 1005 young MSM in Ho Chi Minh City. Most were aged 20 to 24 years (557/1005, 55.4%), highly educated (760/1005, 75.6% with or pursuing a bachelor’s degree or higher), and employed full time (530/1005, 52.7%), and a small proportion had a high income (214/1005, 21.3% making >VND 20 million [US $764.42] per month). Nearly all participants (918/1005, 91.3%) were gay or homosexual. Some reported having ever sold (237/1005, 23.6%) or bought (92/1005, 9.2%) sex. The HIV prevalence was 4.2% (42/1005), with 8.7% (87/1005) reporting unknown status or having never been tested and 17.5% (176/1005) having been diagnosed with an STI in the previous year. Most participants without HIV (624/1005, 62.1%) were on PrEP. Over half had had steady (542/1005, 53.9%) or casual (681/1005, 67.8%) partners in the previous year. More than one-third had >10 male partners (364/1005, 36.2%) and did not know their last partner’s HIV status or had had an HIV-positive last partner (394/1005, 39.2%). A total of 15.8% (159/1005) of the participants had had condomless anal sex without PrEP or ART in their last encounter. Nearly half (444/1005, 44.2%) had participated in group sex. Significant depressive symptoms (428/1005, 42.6%), suicidality (223/1005, 22.2%), hazardous drinking (320/1005, 31.8%), and lifetime illicit sexualized drug use (201/1005, 20%) were common. Most participants (766/1005, 76.2%) had used the internet or apps to find sex partners. Mean scores for perceived HIV stigma, outness, and social support were 10.22 (SD 4.06), 8.36 (SD 6.51), and 3.50 (SD 1.08), respectively. Participant characteristics by patterns of online sex seeking were shown in Table 2.

**Table 1:** Self-reported sociodemographic, behavioral, and psychosocial characteristics of the participants (N=1005).

Variable			Values
**Age (y), n (%)**			
	15-19	76 (7.6)	
	20-24	557 (55.4)	
	25-29	372 (37)	
**Educational level, n (%)**			
	Secondary or lower (≤12 y)	32 (3.2)	
	Tertiary [14,15]	213 (21.2)	
	Bachelor’s degree [16]	622 (61.9)	
	Higher than a bachelor’s degree (≥17 y)	138 (13.7)	
**Employment status, n (%)**			
	Full time	530 (52.7)	
	Not working	184 (18.3)	
	Part time	291 (29)	
**Monthly income, n (%)**			
	<VND 5 million (US $191.11)	164 (16.3)	
	VND 5 million-9,999,999 (US $191.11-$382.21)	254 (25.3)	
	VND 10 million-14,999,999 (US $382.21-$573.31)	220 (21.9)	
	VND 15 million-19,999,999 (US $573.31-$764.42)	153 (15.2)	
	≥VND 20 million (US $764.42)	214 (21.3)	
**Sexual orientation, n (%)**			
	Homosexual	918 (91.3)	
	Bisexual or straight	87 (8.7)	
**Ever sold sex, n (%)**			
	No	768 (76.4)	
	Yes	237 (23.6)	
**Ever bought sex, n (%)**			
	No	913 (90.8)	
	Yes	92 (9.2)	
**HIV status, n (%)**			
	Negative	876 (87.2)	
	Positive	42 (4.2)	
	Unknown	87 (8.7)	
**Any STI^a^ diagnosis (previous 12 mo), n (%)**			
	No	829 (82.5)	
	Yes	176 (17.5)	
**HIV testing (previous 6 mo), n (%)**			
	No	203 (20.2)	
	Yes	802 (79.8)	
**Currently on PrEP^b^, n (%)**			
	No	381 (37.9)	
	Yes	624 (62.1)	
**Steady sex partner (previous 12 mo), n (%)**			
	No	463 (46.1)	
	Yes	542 (53.9)	
**Casual sex partner (previous 12 mo), n (%)**			
	No	324 (32.2)	
	Yes	681 (67.8)	
**Male sex worker partner (previous 12 mo), n (%)**			
	No	964 (95.9)	
	Yes	41 (4.1)	
**Male client sex partner (previous 12 mo), n (%)**			
	No	932 (92.7)	
	Yes	73 (7.3)	
**Number of male sex partners (previous 12 mo), n (%)**			
	1-10	641 (63.8)	
	≥11	364 (36.2)	
**HIV status of last male sex partner, n (%)**			
	Did not know	355 (35.3)	
	HIV positive	39 (3.9)	
	HIV negative	611 (60.8)	
**Condomless anal sex: previous 3 mo, n (%)**			
	No	581 (57.8)	
	Yes	424 (42.2)	
**Condomless anal sex: last time, n (%)**			
	No	583 (58)	
	Yes	422 (42)	
**Condomless anal sex while not on PrEP or ART^c^ (last time), n (%)**			
	No	846 (84.2)	
	Yes	159 (15.8)	
**Group sex (lifetime), n (%)**			
	Never	561 (55.8)	
	1-10 times	335 (33.3)	
	≥11 times	109 (10.8)	
**Frequency of having sex with online partners (previous 12 mo), n (%)**			
	Never	239 (23.8)	
	Sometimes	479 (47.7)	
	Often	287 (28.6)	
**Significant depressive symptoms (CES-D-R^d^ score of >9), n (%)**			
	No	577 (57.4)	
	Yes	428 (42.6)	
**Suicidality (SBQ-R^e^ score of >6), n (%)**			
	No	759 (75.5)	
	Yes	223 (22.2)	
	Missing	23 (2.3)	
**Hazardous drinking (AUDIT-C^f^ score of >3), n (%)**			
	No	685 (68.2)	
	Yes	320 (31.8)	
**Sexualized illicit drug use, n (%)**			
	No	804 (80)	
	Yes	201 (20)	
Perceived HIV stigma (score of 0-21), mean (SD)			10.2 (3.97)
Outness (score of 5-25), mean (SD)			8.36 (6.51)
Social support (MOS-SSS^g^; score of 1-5), mean (SD)			3.50 (1.08)

^a^STI: sexually transmitted infection.

^b^PrEP: preexposure prophylaxis.

^c^ART: antiretroviral therapy.

^d^CES-D-R: Center for Epidemiologic Studies Short Depression Scale.

^e^SBQ-R: Suicide Behaviors Questionnaire–Revised.

^f^AUDIT-C: Alcohol Use Disorders Identification Test–Consumption scale.

^g^MOS-SSS: Medical Outcomes Study Social Support Survey.

**Table 2:** Participant characteristics by patterns of online sex seeking (4-class latent class analysis model).

Variable			Negligible app users (n=638)		Gay app users (n=103)		Poly app users (n=41)		Low-cost app users (n=223)
**Age (y), n (%)**									
	15-19	49 (7.7)		8 (7.8)		4 (9.8)		15 (6.7)	
	20-24	337 (52.8)		70 (68)		27 (65.9)		123 (55.2)	
	25-29	252 (39.5)		25 (24.3)		10 (24.4)		85 (38.1)	
**Educational level, n (%)**									
	Secondary or lower (≤12 y)	22 (3.4)		0 (0)		2 (4.9)		8 (3.6)	
	Tertiary [14,15]	108 (16.9)		33 (32)		17 (41.5)		55 (24.7)	
	Bachelor’s degree [16]	388 (60.8)		67 (65)		20 (48.8)		147 (65.9)	
	Higher than a bachelor’s degree (≥17 y)	120 (18.8)		3 (2.9)		2 (4.9)		13 (5.8)	
**Employment status, n (%)**									
	Full time	312 (48.9)		64 (62.1)		27 (65.9)		127 (57)	
	Not working	146 (22.9)		5 (4.9)		3 (7.3)		30 (13.5)	
	Part time	180 (28.2)		34 (33)		11 (26.8)		66 (29.6)	
**Monthly income, n (%)**									
	<VND 5 million (US $191.11)	101 (15.8)		6 (5.8)		6 (14.6)		51 (22.9)	
	VND 5 million-9,999,999 (US $191.11-$382.21)	168 (26.3)		7 (6.8)		6 (14.6)		73 (32.7)	
	VND 10 million-14,999,999 (US $382.21-$573.31)	133 (20.8)		25 (24.3)		11 (26.8)		51 (22.9)	
	VND 15 million-19,999,999 (US $573.31-$764.42)	87 (13.6)		34 (33)		11 (26.8)		21 (9.4)	
	≥VND 20 million (US $764.42)	149 (23.4)		31 (30.1)		7 (17.1)		27 (12.1)	
**Sexual orientation, n (%)**									
	Homosexual	584 (91.5)		95 (92.2)		33 (80.5)		206 (92.4)	
	Bisexual or straight	54 (8.5)		8 (7.8)		8 (19.5)		17 (7.6)	
**Ever sold sex, n (%)**									
	No	560 (87.8)		25 (24.3)		14 (34.1)		169 (75.8)	
	Yes	78 (12.2)		78 (75.7)		27 (65.9)		54 (24.2)	
**Ever bought sex, n (%)**									
	No	595 (93.3)		93 (90.3)		38 (92.7)		187 (83.9)	
	Yes	43 (6.7)		10 (9.7)		3 (7.3)		36 (16.1)	
**HIV status, n (%)**									
	Negative	552 (86.5)		97 (94.2)		32 (78)		195 (87.4)	
	Positive	24 (3.8)		3 (2.9)		5 (12.2)		10 (4.5)	
	Unknown	62 (9.7)		3 (2.9)		4 (9.8)		18 (8.1)	
**Any STI^a^ diagnosis (previous 12 mo), n (%)**									
	No	537 (84.2)		80 (77.7)		28 (68.3)		184 (82.5)	
	Yes	101 (15.8)		23 (22.3)		13 (31.7)		39 (17.5)	
**HIV testing (previous 6 mo), n (%)**									
	No	123 (19.3)		25 (24.3)		13 (31.7)		42 (18.8)	
	Yes	515 (80.7)		78 (75.7)		28 (68.3)		181 (81.2)	
**Currently on PrEP^b^, n (%)**									
	No	254 (39.8)		14 (13.6)		12 (29.3)		101 (45.3)	
	Yes	384 (60.2)		89 (86.4)		29 (70.7)		122 (54.7)	
**Steady sex partner (previous 12 mo), n (%)**									
	No	272 (42.6)		74 (71.8)		21 (51.2)		96 (43)	
	Yes	366 (57.4)		29 (28.2)		20 (48.8)		127 (57)	
**Casual sex partner (previous 12 mo), n (%)**									
	No	260 (40.8)		7 (6.8)		11 (26.8)		46 (20.6)	
	Yes	378 (59.2)		96 (93.2)		30 (73.2)		177 (79.4)	
**Male sex worker partner (previous 12 mo), n (%)**									
	No	626 (98.1)		95 (92.2)		26 (63.4)		217 (97.3)	
	Yes	12 (1.9)		8 (7.8)		15 (36.6)		6 (2.7)	
**Male client sex partner (previous 12 mo), n (%)**									
	No	615 (96.4)		84 (81.6)		27 (65.9)		206 (92.4)	
	Yes	23 (3.6)		19 (18.4)		14 (34.1)		17 (7.6)	
**Number of male sex partners (previous 12 mo), n (%)**									
	1-10	441 (69.1)		4 (3.9)		9 (22)		187 (83.9)	
	≥11	197 (30.9)		99 (96.1)		32 (78)		36 (16.1)	
**HIV status of last male sex partner, n (%)**									
	Did not know	203 (31.8)		72 (69.9)		27 (65.9)		53 (23.8)	
	HIV positive	32 (5)		1 (1)		1 (2.4)		5 (2.2)	
	HIV negative	403 (63.2)		30 (29.1)		13 (31.7)		165 (74)	
**Condomless anal sex: previous 3 mo, n (%)**									
	No	424 (66.5)		20 (19.4)		8 (19.5)		129 (57.8)	
	Yes	214 (33.5)		83 (80.6)		33 (80.5)		94 (42.2)	
**Condomless anal sex: last time, n (%)**									
	No	410 (64.3)		33 (32)		11 (26.8)		129 (57.8)	
	Yes	228 (35.7)		70 (68)		30 (73.2)		94 (42.2)	
**Condomless anal sex while not on PrEP or ART^c^ (last time), n (%)**									
	No	538 (84.3)		97 (94.2)		35 (85.4)		176 (78.9)	
	Yes	100 (15.7)		6 (5.8)		6 (14.6)		47 (21.1)	
**Group sex (lifetime), n (%)**									
	Never	425 (66.6)		7 (6.8)		2 (4.9)		127 (57)	
	1-10 times	155 (24.3)		68 (66)		21 (51.2)		91 (40.8)	
	≥11 times	58 (9.1)		28 (27.2)		18 (43.9)		5 (2.2)	
**Frequency of having sex with online partners (previous 12 mo), n (%)**									
	Never	239 (37.5)		0 (0)		0 (0)		0 (0)	
	Sometimes	294 (46.1)		9 (8.7)		7 (17.1)		169 (75.8)	
	Often	105 (16.5)		94 (91.3)		34 (82.9)		54 (24.2)	
**Significant depressive symptoms (CES-D-R^d^ score of >9), n (%)**									
	No	388 (60.8)		72 (69.9)		20 (48.8)		97 (43.5)	
	Yes	250 (39.2)		31 (30.1)		21 (51.2)		126 (56.5)	
**Suicidality (SBQ-R^e^ score of >6), n (%)**									
	No	480 (75.2)		98 (95.1)		30 (73.2)		151 (67.7)	
	Yes	141 (22.1)		4 (3.9)		9 (22)		69 (30.9)	
	Missing	17 (2.7)		1 (1)		2 (4.9)		3 (1.3)	
**Hazardous drinking (AUDIT-C^f^ score of >3), n (%)**									
	No	486 (76.2)		50 (48.5)		20 (48.8)		129 (57.8)	
	Yes	152 (23.8)		53 (51.5)		21 (51.2)		94 (42.2)	
**Sexualized illicit drug use, n (%)**									
	No	576 (90.3)		18 (17.5)		15 (36.6)		195 (87.4)	
	Yes	62 (9.7)		85 (82.5)		26 (63.4)		28 (12.6)	
Perceived HIV stigma (score of 0-21), mean (SD)			10.2 (3.81)		7.42 (1.18)		10.7 (6.19)		11.3 (4.15)
Outness (score of 5-25), mean (SD)			8.51 (6.90)		11.2 (4.49)		8.24 (5.43)		6.66 (5.81)
Social support (MOS-SSS^g^; score of 1-5), mean (SD)			3.54 (1.13)		4.09 (0.66)		3.45 (0.90)		3.12 (0.98)

^a^STI: sexually transmitted infection.

^b^PrEP: preexposure prophylaxis.

^c^ART: antiretroviral therapy.

^d^CES-D-R: Center for Epidemiologic Studies Short Depression Scale.

^e^SBQ-R: Suicide Behaviors Questionnaire–Revised.

^f^AUDIT-C: Alcohol Use Disorders Identification Test–Consumption scale.

^g^MOS-SSS: Medical Outcomes Study Social Support Survey.

### App Use and App Use Frequencies for Online Sex Seeking

Table S2 in Multimedia Appendix 1 shows the frequencies and proportions of specific online platform use for sex partner seeking among young MSM in Ho Chi Minh City. A total of 75.9% (763/1005) had used an app for online sex partner seeking. Blued (575/1005, 57.2%) was the most endorsed, followed by Grindr (332/1005, 33%), Facebook (228/1005, 22.7%), Zalo (223/1005, 22.2%), Tinder (181/1005, 18%), Jack’d (162/1005, 16.1%), Twitter (157/1005, 15.6%), Hornet (57/1005, 5.7%), and Line (18/1005, 1.8%). Gay app and poly app users had higher frequencies of online sex seeking, whereas low-cost app users had more frequent online sex seeking than negligible app users but lower frequencies than gay and poly app users.

### LCA Results

Comparing the fit indexes of models with 2 to 6 latent classes (Table S1 in Multimedia Appendix 1), a 4-class model was selected based on the lowest BIC and conditional Akaike information criterion values suggesting the best fit, with an entropy value of 0.82 indicating adequate class separation. The Vuong-Lo-Mendell-Rubin likelihood ratio tests showed that the 4-class model was more informative than the 3- and 5-class models were. Table 3 presents the prevalence and posterior probabilities for the 4–latent class model of online platform use for sex partner seeking among MSM from Ho Chi Minh City. The classes were as follows: *negligible app users* (638/1005, 63.5%), *gay app users* (103/1005, 10.2%), *poly app users* (41/1005, 4.1%), and *low-cost app users* (223/1005, 22.2%). *Negligible app users* endorsed low levels of any app use for sex seeking. *Gay app users* endorsed high levels of gay app use (Grindr, Jack’d, and Blued) and low levels of Tinder, social media, and instant messaging use. *Poly app users* endorsed high levels of use of all apps except Line. *Low-cost app users* endorsed high levels of Blued, Zalo, and Facebook use (Table S2 in Multimedia Appendix 1).

**Table 3:** Associations between patterns of online sex seeking and last instance of condomless anal sex while not on preexposure prophylaxis or antiretroviral therapy stratified by level of social support and outness^a^.

		aPR^b^ (95% CI)
**Model 1a: high social support**		
	Gay app users	0.33 (0.12-0.92)^c^
	Poly app users	0.68 (0.26-1.76)
	Low-cost app users	0.96 (0.64-1.44)
	Negligible app users	Reference
**Model 1b: low social support**		
	Gay app users	0.38 (0.06-2.44)
	Poly app users	1.00 (0.26-3.83)
	Low-cost app users	1.41 (0.93-2.13)
	Negligible app users	Reference
**Model 2a: high outness**		
	Gay app users	0.19 (0.05-0.70)^c^
	Poly app users	0.21 (0.02-1.85)
	Low-cost app users	1.33 (0.80-2.22)
	Negligible app users	Reference
**Model 2b: low outness**		
	Gay app users	0.46 (0.07-2.99)
	Poly app users	1.00 (0.30-3.36)
	Low-cost app users	1.47 (0.99-2.18)^d^
	Negligible app users	0.46 (0.07-2.99)

^a^High social support and high outness were defined as equal to or above the sample means. Models 1a and 1b were adjusted for educational level, employment status, income, whether participants had ever sold sex, steady partnership, any sexually transmitted infection (STI) diagnoses, condom use in the previous 3 months, outness, and HIV stigma. Models 2a and 2b were adjusted for educational level, employment status, income, whether participants had ever sold sex, steady partnership, any STI diagnoses, condom use in the previous 3 months, and HIV stigma.

^b^aPR: adjusted prevalence ratio.

^c^Significant at *P*<.05.

^d^Marginally significant, with a *P* value between .05 and .06.

### Characterization of Sociodemographic, Sexual Behavioral, and Psychosocial Correlates With Patterns of Online Platform Use for Sex Seeking

Latent class membership was characterized using crude rates, means, SDs (Table 4), univariate logistic regression (Table S3 in Multimedia Appendix 1), multivariable multinomial logistic regressions (Table 4), and sets of theoretically relevant characteristics (Table S4 in Multimedia Appendix 1). In the multivariable model (Table 4), compared to *negligible app users*, *gay app users* were more likely to have casual sex partners, more male sex partners, group sex experience, and lower perceived HIV stigma, as well as engage in heavy drinking and sexualized illicit drug use. Sexualized illicit drug use best characterized gay app users. *Poly app users* were more likely to have casual sex partners, male sex worker partners, male client sex partners, and more male sex partners and engage in sexualized illicit drug use. Having male sex worker partners best characterized poly app users. *Low-cost app users* were more likely to have lower incomes, casual sex partners, male client sex partners, lower social support, and higher perceived HIV stigma. Having casual sex partners best characterized low-cost app users. In Table S4 in Multimedia Appendix 1, sexual partnerships best predicted (through the BIC) the difference in online sex-seeking patterns, followed by socioeconomic status and sexual health indicators. However, substance use best explained the difference (adjusted *R*^2^), followed by sexual health indicators and sexual partnerships.

**Table 4:** Multivariable multinominal logistic regression models examining characteristics associated with latent class memberships (reference group: negligible app users).

Variable			Gay app users				Poly app users					Low-cost app users		
			aRRR^a^ (95% CI)		RI^b^ (%)		aRRR (95% CI)		RI (%)		aRRR (95% CI)			RI (%)
**Monthly income**														
	<VND 10 million (US $382.21)	0.50 (0.23-1.10)		1.27		1.45 (0.61-3.44)		0.08		*1.57 (1.12-2.19)* ^c^			1.32	
	≥VND 10 million (US $382.21)	Reference		—^d^		Reference		—		Reference			—	
**Casual sex partners (previous 12 mo)**														
	Yes	*7.66 (2.49-23.6)*		1.36		*4.42 (1.40-13.9)*		0.03		*3.33 (2.25-4.92)*			2.73^e^	
	No	Reference		—		Reference		—		Reference			—	
**Male sex worke** **r** **partner (p** **revious** **12 mo** **)**														
	Yes	1.78 (0.48-6.65)		0.24		*14.9 (4.80-46.5)*		7.88^e^		1.68 (0.57-4.98)			0.05	
	No	Reference		—		Reference		—		Reference			—	
**Male client sex partner (p** **revious** **12 mo** **)**														
	Yes	2.68 (0.95-7.55)		0.59		*3.88 (1.34-11.3)*		1.97		*2.39 (1.16-4.94)*			0.23	
	No	Reference		—		Reference		—		Reference			—	
Number of male sex partners (previous 12 mo)			*1.01 (1.00-1.02)*		3.49		*1.02 (1.01-1.03)*		5.32		0.98 (0.97-1.00)			1.34
**Ever had group sex**														
	>10 times	*3.36 (1.67-6.74)*		8.50		2.36 (0.97-5.78)		1.37		0.63 (0.37-1.09)			1.44	
	<10 times	Reference		—		Reference		—		Reference			—	
**Hazardous alcohol use**														
	Yes	*2.74 (1.43-5.26)*		0.87		1.74 (0.79-3.85)		0.22		*2.35 (1.66-3.32)*			1.71	
	No	Reference		—		Reference		—		Reference			—	
**Ever engaged in sexualized illicit drug use**														
	Yes	*14.4 (7.38-28.1)*		16.35^e^		*5.78 (2.49-13.4)*		1.96		1.16 (0.68-1.97)			0.49	
	No	Reference		—		Reference		—		Reference			—	
Social support			1.19 (0.80-1.79)		1.23		0.71 (0.45-1.13)		0.28		*0.83 (0.69-0.99)*			1.74
Perceived HIV stigma			*0.72 (0.60-0.86)*		2.95		0.98 (0.86-1.12)		0.07		*1.05 (1.00-1.10)*			1.56

^a^aRRR: adjusted relative risk ratio.

^b^RI: relative importance weight percentage—proportion of *R*^2^ contributed by each covariate. A higher percentage indicates a higher contribution to explaining the differences in patterns of online sex seeking.

^c^Italicized values are significant at *P*<.05.

^d^Not applicable.

^e^Highest relative importance weight percentage among the latent class subgroup.

### Moderating Relationships Among Social Support, Patterns of Online Sex Seeking, and Last Instance of Condomless Anal Sex Without PrEP or ART

Gay app use was independently associated with a lower prevalence of condomless anal sex without PrEP or ART in their last sexual encounter regardless of confounding adjustments, social support, and outness levels and their moderating effects. A significant 3-way moderating relationship (*P*=.01) was found among social support, outness, and online sex-seeking patterns on last instance of condomless anal sex without PrEP or ART (Table S5 in Multimedia Appendix 1). Stratified analyses by high and low levels of social support and outness (stratified by sample means) are reported in Table 3. Gay app use was significantly associated with a lower prevalence of condomless anal sex without PrEP or ART in their last sexual encounter among participants with high levels of social support and outness. Among those with low outness, negligible app use was marginally associated with a higher prevalence of condomless anal sex without PrEP or ART in their last sexual encounter.

## Discussion

### Principal Findings

To our knowledge, this is the first study to identify and examine nuanced patterns of online sex seeking among MSM from Southeast Asia. Our study is also among the first to report quantitative survey data using a robust sample size (N=1005) of MSM from Vietnam with an optimal response rate (1005/1270, 79.1%) [4,67-72], and among the few that recruited young MSM from Vietnam (aged 15-29 years) [73]. In our sample of mostly gay-identified young MSM from Vietnam, we identified 4 distinct subgroups of young MSM based on the social networking apps they self-reported actively using for sex seeking: (1) *negligible app users*, (2) *gay app users*, (3) *poly app users*, and (4) *low-cost app users*. Using different modeling approaches, a consistent pattern emerged in identifying the one characteristic that best described the difference in patterns of online sex seeking, which supports our hypothesis and is consistent with findings that MSM networks and online social networks in general tend to be homophilic (similar) in size (number of partners) and partnership types [25,59,74]. Participant age was the variable that predicted and explained the difference in patterns of online sex seeking to the lowest extent. This is consistent with network findings that young MSM users of the largest Asia-based gay dating app, Blued, tend to develop relationships MSM older than themselves (disassortative on age) [75]. Thus, participants’ ages were less homogenous across patterns of online sex seeking. As indicated by the relative importance weight (a decomposition of the explained variance of the model) in the multivariable multinomial model, gay app users were most distinct in reporting sexualized illicit drug use (relative importance=16.35%), which is a homophilic behavior within MSM’s online social networks [17,44]. Wong et al [76] found that, compared to non–chemsex-engaged MSM, chemsex-engaged MSM in Hong Kong had higher degrees of online social network centrality and closeness, which are characteristics positively associated with social support [59]. This is similar to how gay app users in our survey, despite reporting the highest burden of sexualized illicit drug use, also had the highest level of social support and were the least stigmatized.

Multiple modeling approaches converged to indicate sexual partnerships as the most salient similarity among poly app users and the most distinctive difference in patterns of online sex seeking. The grouping of poly app users could underly sexual network similarities and closeness, which are strongly predictive of their elevated HIV and STI prevalence (5/41, 12% and 13/41, 32%, respectively) and affiliations with a higher number of sexual networks [25-27]. These attributes suggest the role of poly app users in bridging HIV or STI transmissions among online platforms, groups, partner types, and networks [25]. At the same time, poly app users accounted for only 4.1% (41/1005) of the total sample size but had the highest prevalence of HIV and STI diagnoses. This finding is consistent with the fact that a small number of bridging individuals in HIV transmission networks contribute to large clusters of infections [77]. This is alarming given the currently low ART coverage (32.1%) among MSM living with HIV in Vietnam and the high proportion of undiagnosed HIV infections [72,78]. Although most poly app users reported current ART or PrEP use, their heightened concurrent mental health burdens, sexualized illicit drug use, and heavy drinking could lead to suboptimal adherence [79]. A similar pattern was also found among MSM in central Kentucky, with multiple app users having higher HIV and STI prevalence than other app users [38]. This finding reflects the ubiquitous nature of online social structures. At the same time, young MSM’s multiple app use could be indicative of socially patterned behaviors such as the syndemic production of sexual compulsivity, which was separately shown to be mediated by online sex seeking to increase sexual risk taking [80,81]. Teixeira da Silva et al [82] found that US-based Black MSM and women’s social networks could moderate the syndemic production of HIV vulnerability and other structural determinants. Future studies should investigate the relationship between multiple app use for online sex seeking and the syndemic production of sexual compulsivity [80], as well as the roles of young MSM’s online social network features in moderating and mediating individual HIV vulnerability factors that increase MSM’s probability of HIV seroconversion.

We found that patterns of online sex seeking had a significant independent relationship with condomless anal sex while not on PrEP or ART. Gay app use moderated the protective effects of social support and outness on condomless anal sex, which is suggestive of conjunctive positive condom use and PrEP uptake norms among young MSM who use gay apps exclusively. First, this moderating protective effect specific to gay app users could underlie the success of gay app–focused marketing strategies from HIV prevention programs in Vietnam [83]. However, it is unclear why increasing levels of social support and outness did not have the same protective effects on condomless anal sex as on other patterns of app use for sex seeking. Reback et al [84] found that consistent condom use among transgender women who had a smaller social network size and density (indicating lower social support) was more strongly influenced by cisgender partners who interacted with them online than by other transgender women with more social support. This suggests a direct social influence without being linked by a physical sex act, in which participants with lower social support would be more susceptible to social influences on sexual risk taking when exposed to online interactions. Our results indicate that this might occur less frequently on gay dating apps than on other platforms, as demonstrated by the independent protective effect on condomless anal sex while not on PrEP or ART among gay app users. Our findings do not support the hypothesis that passive exposure to online contexts with sexually explicit features (eg, naked photos, profile descriptions about barebacking [condomless anal sex], or drug use practices, which are more enabled and prevalent on gay apps than on other platforms) leads to more sexual risk-taking intentions and behaviors [17,85]. However, passive exposure to online norms could be more relevant to substance use than to condom use behaviors as gay app users in our study also had the highest burden of sexualized illicit drug use [86].

Only exclusive gay app users benefited from their exposure to the online social environment in terms of social support and outness, in contrast to other users, who tended to use mainstream social media platforms. There could be distinctive differences in the social environments and underlying online social networks that young MSM are exposed to when using social media platforms [31]. Young et al [87] found that young Black MSM belonging to Facebook groups that discussed lesbian, gay, bisexual, transgender, and queer identity were less likely to have condomless anal sex than those belonging to other group categories. There are distinct communities and online groups embedded within social media platforms that share similar sexual interests and identities. Exposure to these social structures could interfere with the protective effects of preexisting levels of social support and outness on condomless anal sex and PrEP uptake. Schnarrs et al [88] found that US-based MSM within a single social networking app who identified with different gay subculture labels (eg, “bear” or “chub”; “muscle” or “jock”; or “pox,” “leather,” or “daddy”) had significant differences in terms of HIV prevalence, PrEP uptake, and condom use. “Hifun” (high and fun) or “bbcf” (barebacking [condomless anal sex] while using “chemsex” or “chemfun”) are some of the popular online labels used among MSM in Asia to indicate their intended sexual purposes, including group sex and sexualized drug use [89]. Our team identified >100 online groups on Facebook and Twitter, among other platforms, each with 50 to 100 MSM members with common sexual interests such as barebacking (condomless anal sex), “chemsex” or sexualized drug use, and group sex in Bangkok, Thailand. Similar online groups could exist in Vietnam and could play a role in the accumulation of HIV vulnerability among young Vietnamese MSM.

Low-cost app users were marginally more likely to have condomless anal sex while not on PrEP or ART when their level of outness was low. The predisposition of low-cost app users to being less out and having less social support could coincide with having a less supportive offline social network, for example, lack of support from family and friends [31]. Despite having similar educational achievements as negligible app users, low-cost app users tended to earn a lower monthly income, indicating a lower socioeconomic status, which is often accompanied by a less functioning social network [59]. Their HIV vulnerability could be further exacerbated by their online social network’s homophilic clustering of mental health burdens and hazardous alcohol drinking [46,59,90]. Low-cost app users’ access to gay dating apps (eg, Grindr, Jack’d, and Hornet) could also be limited by their increased economic strain as most gay dating apps have higher premium prices (which are lower for Blued) to access more app features and a higher number of other users’ profiles. This lack of access could also extend to their engagement with NGOs and HIV-preventive services in Vietnam because these organizations heavily use gay apps as advertising partners, as evidenced by the lower PrEP uptake compared to other patterns of online sex seeking among this group [91]. HIV prevention programs in Vietnam should diversify their outreach focus to include Blued and design and implement tailored online outreach strategies to address the psychosocial clustering of HIV vulnerability among young MSM of lower socioeconomic statuses who may have limited access to MSM-related resources.

From an ecological perspective, social networking apps can also be conceptualized by users’ interactions with each app’s features [39]. The adoption of HIV- or STI-preventive strategies was postulated to be enabled by MSM’s use of sexual health features on gay apps, for example, the facilitation of seroadaptive behaviors or biomedical matching based on partners’ sexual health profiles (eg, PrEP status) [22,23]. Blued is the only gay app included in our study that does not include any sexual health features on user profiles. However, there is a higher crude rate of condomless anal sex (with or without PrEP or ART) among Blued users than among users of other gay apps. After adjusting for sociodemographic and psychosocial characteristics, Blued users were significantly less likely to have condomless anal sex than those who did not use Blued. A similar protective effect was not found for other gay apps (Grindr, Jack’d, and Hornet). Therefore, it is unclear how sexual health features might have facilitated HIV risk-mitigating behaviors among young Vietnamese MSM in our study. While 67% of surveyed American MSM who use gay apps report using one of these sexual health features [24], it is unknown who among young Southeast Asian MSM use these features and how. Future studies should investigate the use of these sexual health features among young Southeast Asian MSM and the role of sociocultural influences on the perceptions of these features in facilitating the adoption of effective HIV risk-mitigating behaviors such as PrEP use and HIV testing.

### Implications

Our study shows that young Vietnamese MSM have nuanced online sex-seeking patterns (or grouping), and these patterns reflect the complexity of their access to and engagement with social networking apps, reflecting the varying degrees of exposure to online social structures and social influences on young MSM’s HIV vulnerability. However, existing social networking app–based interventions primarily focus on micro-level behavioral factors, neglecting the social structural influences that could moderate the effectiveness of these online interventions [92]. Our study demonstrates that the protective effect of social support and outness on a high probability of HIV seroconversion behavior (condomless anal sex on their last sexual encounter while not on PrEP or ART) could be affected by young MSM’s patterns of online sex seeking in different directions. Digital interventions tailored for young MSM could benefit from understanding how targeted protective or resilient behavior (eg, PrEP uptake) could be affected by young MSM’s patterns of online sex seeking. On the other hand, social network studies could benefit from understanding the patterns of young MSM’s online sex seeking when identifying different online environments as sources of social influence beyond partnership ties. Collectively, our study augments the emerging literature by refuting the assumption that all MSM engaged in online sex seeking belong to one homogeneous group independent of preexisting social conditions and influences [36-38,88,93].

### Limitations

Despite our study’s novel contribution to the existing literature, it has some limitations. This study used a convenience sample of moderately well-educated and high-income young MSM from Ho Chi Minh City, which may not represent the underlying young MSM population in Vietnam. In our sensitivity analyses using split samples, the confounder-adjusted associations were robust to random resampling with at least a 70% cutoff, providing additional confidence against false detection due to sampling bias [94]. Compared with previous studies among MSM in Vietnam, often recruited through respondent-driven sampling, our participants were less likely to report being bisexual or heterosexual and were less likely to have had previous sex work involvement but had similar rates of STI prevalence, drug use, hazardous alcohol use, and depression [4,69]. The prevalence of online sex-seeking behaviors in our sample (766/1005, 76.2%) was similar to that found in a previous study (76.5%) [7]. Nevertheless, our participants had a lower prevalence of condomless anal intercourse in their last sexual encounter than that in the aforementioned study (422/1005, 42% vs 63%) [7]. Therefore, if our participants’ rate of condomless anal sex was higher, such as those in another study or among the underlying population [7], our current estimations of the associations between condomless anal sex and patterns of app use would have been underestimated due to differential misclassification (equal exposure: online sex seeking; unequal outcome: condomless anal sex). Similarly, the prevalence of HIV-preventive strategies (HIV testing and PrEP uptake) and their associations with patterns of online sex seeking were likely overestimated, with the assumption that higher rates of PrEP uptake and HIV testing were estimated than in the underlying true population. As expected, data from this study were self-reported and, therefore, subject to recall and social desirability biases. Using an online self-administered survey could minimize this social desirability bias. The cross-sectional design of this study limits the interpretability of the results. However, according to conventional standards, we accounted for a comprehensive list of potential confounders and minimized the adjustments for potential mediators [63]. A longitudinal design is preferred to confirm the causality of the associations identified in our study. Consistent with other studies that have used LCA, proper class assignment was not guaranteed, but quality assurance indexes indicated that the optimal conditions were met. The qualitative and subjective naming of the latent classes may not accurately reflect the data structure when determining latent class memberships. However, the consistency of app use patterns identified in another study with MSM from the United States provided some confidence in our study’s external validity and labeling of latent class assignment [38].

### Conclusions

Our findings show that there is a high level of complexity in young Vietnamese MSM’s patterns of social networking app use for sex seeking. Those who exclusively used gay apps (*gay app users*) engaged in more optimal HIV-preventive practices and had more optimal psychosocial statuses than those with other patterns of online sex seeking. Higher levels of social support and outness among gay app users were associated with a lower prevalence of condomless anal sex in their last sexual encounter while not on PrEP or ART (high-probability HIV seroconversion behavior). However, young MSM who use multiple apps for sex seeking (*poly app users*) and those who only use apps that are free (social media) or with a lower price threshold (Blued) did not benefit from the protective effects of social support and outness to the same extent. *Low-cost app users* with a lower level of outness were marginally more likely to engage in risky sexual behaviors than young MSM who did not use apps for sex seeking (*negligible app users*). The higher crude rates for high-probability HIV seroconversion behaviors of poly app users and low-cost app users could be attributed to their potential exposure to different online social environments and underlying social networks. These exposures could also contribute to the clustering of psychosocial adversities among poly app users and low-cost app users. Future studies should disentangle the sources, levels, and degrees of social influence on young MSM’s HIV vulnerability by understanding the relationship between young MSM’s complex patterns of online sex seeking and their respective exposures to different social structures and influences.

## Appendix

Multimedia Appendix 1 Supplementary tables of analyses from a sample of young, gay, bisexual and other men who have sex with men from Ho Chi Minh City, Vietnam.

## Abbreviations

ART: antiretroviral therapy
BIC: Bayesian information criterion
LCA: latent class analysis
MSM: men who have sex with men
NGO: nongovernmental organization
PrEP: preexposure prophylaxis
STI: sexually transmitted infection

## Data Availability

The datasets generated or analyzed during this study are available from the corresponding author on reasonable request.
