# Supplementary material for: Sexual and Psychosocial Risk Burdens Associated With Online Sex Seeking Among Young Men Who Have Sex With Men: Cross-Sectional Study
Source: J Med Internet Res. 2025 Aug 20;27:e59072. doi: 10.2196/59072 (PMC12409178; doi:10.2196/59072)
Supplement: Multimedia Appendix 1 [file jmir_v27i1e59072_app1.docx]

| Supplementary Table 1. Goodness-of-fit indices for model selection and model diagnostic | | | | | | | | |
| --- | --- | --- | --- | --- | --- | --- | --- | --- |
| Model | df | LL | G^2^ | AIC | BIC | CAIC | Χ^2^ | Entropy |
| 2-class | 492 | -3694.03 | 828.94 | 7426.06 | 7519.40 | 7538.40 | 3894.02 | 0.74 |
| 3-class | 482 | -3539.47 | 519.82 | 7136.94 | 7279.41 | 7308.41 | 3115.7 | 0.80 |
| 4-class^a^ | 472 | -3405.83 | 252.54 | 6889.67 | 7081.26 | 7120.26 | 627.76 | 0.82 |
| 5-class | 462 | -3393.60 | 228.08 | 6885.21 | 7125.93 | 7174.93 | 588.43 | 0.90 |
| 6-class | 452 | -3381.66 | 204.20 | 6881.33 | 7171.18 | 7230.18 | 550.40 | 0.89 |
| **Model parameters:**  df: residual degrees of freedom  LL: log-likelihood.  G^2^: likelihood ratio test statistic.  **Model fit indices:**  AIC: Aikaike information criterion.  BIC: Bayesian information criterion.  CAIC: conditional Aikaike information criterion.  Χ^2^: chi-square goodness of fit test statistic.  **Model diagnostic:**  Entropy: a value higher than 0.80 is defined as having an adequate separation between latent classes  ^a^Selected class based on lowest BIC and CAIC values. | | | | | | | | |

| Supplementary Table 2. Frequency and proportion of specific app use, proportion of recurrent use and posterior probabilities by latent class membership | | | | | | | | | |
| --- | --- | --- | --- | --- | --- | --- | --- | --- | --- |
|  | Total | Negligible app users  (63.5%, n=638) | | Gay app users  (10.2%, n=103) | | Poly app users  (4.1%, n=41) | | Low cost app users (22.0%, n=223) | |
|  | N (%) | (%)* | PP | (%)* | PP | (%)* | PP | (%)* | PP |
| **Hornet** | 57 (5.7) | (1.4) | 0.03 | (8.7) | 0.09 | (53.7) | 0.65 | (0) | 0.01 |
| **Jackd** | 162 (16.1) | (0.5) | 0.03 | (89.3) | 0.76 | (82.9) | 0.99 | (1.8) | 0.04 |
| **Grindr** | 332 (33.0) | (7.5) | 0.17 | (91.3) | 1.00 | (82.9) | 0.97 | (7.6) | 0.27 |
| **Blued** | 575 (57.2) | (10.5) | 0.38 | (84.5) | 0.94 | (82.9) | 1.00 | (23.3) | 0.78 |
| **Tinder** | 181 (18.0) | (2.8) | 0.10 | (5.8) | 0.15 | (65.9) | 0.83 | (5.8) | 0.29 |
| **Twitter/X** | 157 (15.6) | (0.2) | 0.02 | (15.5) | 0.15 | (82.9) | 1.00 | (14.3) | 0.35 |
| **Facebook** | 228 (22.7) | (0.3) | 0.07 | (2.9) | 0.03 | (70.7) | 0.91 | (16.6) | 0.60 |
| **Zalo** | 223 (22.2) | (0) | 0.00 | (0) | 0.01 | (68.3) | 0.87 | (21.1) | 0.76 |
| **Line** | 18 (1.8) | (0) | 0.00 | (0) | 0.00 | (12.2) | 0.26 | (0.4) | 0.03 |
| **Any app** | 763 (75.9) | (16.5) | -- | (91.3) | -- | (82.9) | -- | (24.2) | -- |
| *Proportion of participants reporting both “often” meeting sex partners online and specific app use for sex-seeking  PP=Posterior probabilities estimated from latent class analysis (4-class model) | | | | | | | | | |

| Supplementary Table 3. Bivariate multinominal logistic regression models examining characteristics associated with latent class memberships (reference group = negligible app user) | | | | | | |
| --- | --- | --- | --- | --- | --- | --- |
|  | Gay app users | | Poly app users | | Low cost app users | |
| Variable | OR | (95% CI) | OR | (95% CI) | OR | (95% CI) |
| **Age** |  |  |  |  |  |  |
| 15-19 | 1.65 | (0.70-3.86) | 2.57 | (0.62-6.82) | 0.91 | (0.48-1.70) |
| 20-24 | **2.09** | **(1.29-3.40)** | 2.01 | (0.96-4.25) | 1.08 | (0.78-1.49) |
| 25-29 | Ref |  | Ref |  | Ref |  |
| **Education** |  |  |  |  |  |  |
| Tertiary or below | **10.15** | **(3.03-33.97)** | **8.77** | **(2.00-38.44)** | **4.47** | **(2.34-8.53)** |
| Bachelor degree | **6.91** | **(2.13-22.35)** | 3.09 | (0.71-13.42) | **3.45** | **(1.91-6.39)** |
| Higher than bachelor degree | Ref |  | Ref |  | Ref |  |
| **Employment status** |  |  |  |  |  |  |
| Part-time | 0.92 | (0.58-1.45) | 0.71 | (0.34-11.46) | 0.90 | (0.64-1.28) |
| Not working or student | **0.17** | **(0.07-0.42)** | **0.24** | **(0.07-0.80)** | **0.50** | **(0.32-0.79)** |
| Full-time | Ref |  | Ref |  | Ref |  |
| **Monthly income** |  |  |  |  |  |  |
| <5 million VND | **0.29** | **(0.11-0.71)** | 1.26 | (0.41-3.87) | **2.79** | **(1.64-4.74)** |
| 5 million to 9,999,999 VND | **0.20** | **(0.09-0.47)** | 0.76 | (0.25-2.31) | **2.40** | **(1.46-3.93)** |
| 10 million to 14,999,999 VND | 0.9 | (0.51-1.61) | 1.76 | (0.66-4.67) | **2.11** | **(1.26-3.57)** |
| 15 million to 19,999,999 VND | **1.88** | **(1.08-3.27)** | **2.69** | **(1.01-7.20)** | 1.33 | (0.71-2.50) |
| ≥ 20 million VND | Ref |  | Ref |  | Ref |  |
| **Sexual Orientation** |  |  |  |  |  |  |
| Gay or homosexual | Ref |  | Ref |  | Ref |  |
| Bisexual or straight | 0.91 | (0.42-1.97) | **2.62** | **(1.15-5.96)** | 0.89 | (0.51-1.57) |
| **Ever provided sex work** |  |  |  |  |  |  |
| Yes | **22.4** | **13.47-37.26** | **13.85** | **6.96-27.54** | **2.29** | **1.56-3.38** |
| No | Ref |  | Ref |  | Ref |  |
| **Ever received sex work** |  |  |  |  |  |  |
| Yes | 1.49 | (0.72-3.06) | 1.09 | (0.32-3.58) | **2.66** | **(1.66-4.27)** |
| No | Ref |  | Ref |  | Ref |  |
| **HIV status** |  |  |  |  |  |  |
| Positive | 0.71 | (0.21-2.41) | **3.59** | **(1.29-10.04)** | 1.18 | (0.55-2.51) |
| Unknown or never tested | **0.28** | **(0.08-0.89)** | 1.11 | (0.38-3.25) | 0.82 | (0.47-1.42) |
| Negative | Ref |  | Ref |  | Ref |  |
| **Any STI in the past 12 months** |  |  |  |  |  |  |
| Yes | 1.53 | (0.92-2.55) | **2.47** | **(1.24-4.93)** | 1.13 | (0.75-1.69) |
| No | Ref |  | Ref |  | Ref |  |
| **HIV testing in the past 6 months** |  |  |  |  |  |  |
| Yes | 0.75 | (0.46-1.22) | 0.51 | (0.26-1.02) | 1.03 | (0.70-1.52) |
| No | Ref |  | Ref |  | Ref |  |
| **Currently on PrEP** |  |  |  |  |  |  |
| Yes | **4.20** | **(2.34-7.55)** | 1.60 | (0.80-3.19) | 0.80 | (0.59-1.08) |
| No | Ref |  | Ref |  | Ref |  |
| **Number of male sex partners in the past 12 months** | | | | | | |
| 11+ | **21.03** | **(12.17-36.32)** | **8.59** | **(4.37-16.88)** | **0.44** | **(0.27-0.73)** |
| 1-10 | Ref |  | Ref |  | Ref |  |
| **Condomless anal sex during last sexual encounter** | | | | | | |
| Yes | **3.81** | **(2.45-5.95)** | **4.90** | **(2.41-9.97)** | 1.31 | (0.96-1.79) |
| No | Ref |  | Ref |  | Ref |  |
| **HIV status of last male sex partner** | | | | | | |
| HIV-positive or don’t know | **4.17** | **(2.65-6.57)** | **3.69** | **(1.88-7.27)** | **0.60** | **(0.43-0.85)** |
| HIV-negative | Ref |  | Ref |  | Ref |  |
| **Ever had group sex** |  |  |  |  |  |  |
| 5 times or above | **18.50** | **(11.10-30.80)** | **10.25** | **(5.20-20.23)** | **0.61** | **(0.38-0.99)** |
| Below 5 times or never | Ref |  | Ref |  | Ref |  |
| **Ever had sexualized illicit drug use** |  |  |  |  |  |  |
| Yes | **43.87** | **(24.76-77.73)** | **16.1** | **(8.10-32.02)** | 1.33 | (0.83-2.14) |
| No | Ref |  | Ref |  | Ref |  |
| **Condomless anal sex during last sexual encounter without biomedical prevention strategies** | | | | | | |
| Yes | **0.33** | **(0.14-0.78)** | 0.92 | (0.38-2.25) | 1.44 | (0.98-2.11) |
| No | Ref |  | Ref |  | Ref |  |
| **Hazardous alcohol use** |  |  |  |  |  |  |
| **Yes** | **3.39** | **(2.21-5.20)** | **3.36** | **(1.77-6.36)** | **2.33** | **(1.69-3.22)** |
| **No** | Ref |  | Ref |  | Ref |  |
| **Significant depressive symptoms (CESD-R >9)** |  |  |  |  |  |  |
| Yes | 0.67 | (0.43-1.05) | 1.63 | (0.87-3.07) | **2.02** | **(1.48-2.75)** |
| No | Ref |  | Ref |  | Ref |  |
| **Suicidal** |  |  |  |  |  |  |
| Yes | **0.14** | **(0.05-0.38)** | 1.02 | (0.47-2.20) | **1.56** | **(1.11-2.19)** |
| No | Ref |  | Ref |  | Ref |  |
| **Perceived HIV stigma** | **0.57** | **(0.47-0.69)** | 1.03 | (0.95-1.12) | **1.06** | **(1.03-1.10)** |
| **Social Support** | **1.72** | **(1.38-2.15)** | 0.92 | (0.69-1.23) | **0.69** | **(0.60-0.78)** |
| RRR: relative risk ratio  CI: confidence interval  Ref: reference group | | | | | | |

| Supplementary Table 4. Models grouped by theoretically relevant variables in characterizing patterns of online sex-seeking | | | | | |
| --- | --- | --- | --- | --- | --- |
| Model | df | Adjusted R^2^ | Adjusted R^2^/df | BIC | BIC/df |
| **Age** | 9.00 | 0.6% | 0.07% | 2032.10 | 225.79 |
| **Socioeconomic status** | 27.00 | 8.5% | 0.31% | **1998.99** | **74.04** |
| **Sexual identity** | 9.00 | 2.0% | 0.22% | 2004.33 | 222.70 |
| **Sexual health indicators** | 21.00 | **18.3%** | **0.87%** | **1763.70** | **83.99** |
| **HIV prevention uptake** | 9.00 | 2.9% | 0.32% | 1984.95 | 220.55 |
| **Sexual Partnership** | 27.00 | **21.5%** | **0.80%** | **1740.50** | **64.46** |
| **Substance use** | 9.00 | **15.5%** | **1.72%** | 1735.80 | 192.87 |
| **Mental health** | 9.00 | 4.9% | 0.54% | 1945.14 | 216.13 |
| **Psychosocial status** | 9.00 | 6.9% | 0.77% | 1907.22 | 211.91 |
| df: degrees of freedom  Adjusted R^2^: variation in predicted outcome explained by included variables, adjusted for numbers of insignificant variables in the model; the higher the better.  BIC: Bayesian information criteria – efficiency of the included variables in predicting the outcome, the lower the better. | | | | | |

| Supplementary Table 5: Models to examine the moderating effects of online sex-seeking patterns on last condomless anal sex without PrEP or ART | | |
| --- | --- | --- |
|  | **PR (95% CI)** | **aPR (95% CI)** |
| **Models without social support and outness** | Model 1a | Model 2a |
| Gay app users | 0.37 (0.17-0.83)* | 0.28 (0.12-0.66)* |
| Poly app users | 0.93 (0.44-2.00) | 0.72 (0.32-1.63) |
| Low cost app users | 1.34 (0.98-1.84) | 1.21 (0.90-1.62) |
| Negligible app users | Ref | Ref |
|  |  |  |
| **Models with main effects only** | Model 1b | Model 2b |
| Outness | 0.95 (0.93-0.98)** | 0.97 (0.95-1.00)* |
| Social support | 0.82 (0.70-0.95)* | 0.84 (0.73-0.97)* |
| Gay app users | 0.49 (0.22-1.11) | 0.34 (0.14-0.84)* |
| Poly app users | 0.94 (0.45-1.95) | 0.74 (0.34-1.62) |
| Low cost app users | 1.18 (0.87-1.61) | 1.15 (0.86-1.54) |
| Negligible app users | Ref | Ref |
|  |  |  |
| **Models with main effects and interactions** |  |  |
| Main effects | Model 1c | Model 2c |
| Outness | 1.03 (0.94-1.13) | 0.00 (0.00-6.63) |
| Social support | 0.98 (0.77-1.26) | 0.07 (0.00-1.87) |
| Gay app users | 0.00 (0.00-25.9) | 1.91 (0.44-8.30) |
| Poly app users | 0.11 (0.00-4.58) | 0.97 (0.88-1.06) |
| Low cost app users | 2.43 (0.47-12.5) | 0.89 (0.69-1.15) |
| Negligible app users | Ref | Ref |
|  |  |  |
| **Main effects and 3-way interaction** |  |  |
| Gay app users* outness | 2.51 (1.01-6.25)* | 2.22 (0.98-5.03)* |
| Poly app users* outness | 2.43 (1.49-3.96)** | 2.61 (1.57-4.32)** |
| Low cost app users* outness | 0.90 (0.73-1.09) | 1.00 (0.83-1.22) |
|  |  |  |
| Gay app users* social support | 7.44 (0.52-106) | 6.19 (0.72-53.1) |
| Poly app users* social support | 2.96 (0.71-12.3) | 3.35 (0.88-12.7) |
| Low cost app users* social support | 0.79 (0.46-1.35) | 0.84 (0.52-1.36) |
| Outness* social support | 0.98 (0.95-1.00) | 1.00 (0.97-1.03) |
|  |  |  |
| Gay app users*outness* social support | 0.78 (0.63-0.98)* | 0.80 (0.65-0.98)* |
| Poly app users*outness * social support | 0.68 (0.55-0.85)** | 0.66 (0.52-0.83)** |
| Low cost app users*outness * social support | 1.04 (0.97-1.10) | 1.00 (0.94-1.06) |
| *Significant at <0.05  **Significant at <0.001  Model 2a to 2b: adjusted for education, employment status, income, sold sex, steady partnership, any STI diagnosis, CAS in past 3months  R^2^ Nagelkerke (total variance explained) for models 1a to 2c: 0.027, 0.082, 0.115, 0.299, 0.318, 0.344 | | |

| Supplementary Table 6: Models to examine the moderating effects of online sex-seeking patterns on last condomless anal sex without PrEP or ART excluding post-graduate students (n=969) | |
| --- | --- |
|  | **PR (95% CI)** |
| **Main effects** |  |
| Negligible app users | Ref |
| Gay app users | 0.00 (0.00 – 4.24) |
| Poly app users | 0.06 (0.00 – 1.54) |
| Low cost app users | 1.36 (0.35 – 5.27) |
| Outness | 0.96 (0.87 – 1.06) |
| Social support | 0.85 (0.66 – 1.09) |
| **Main effects and 3-way interaction** |  |
| Gay app users* outness | 2.3 (1.00 – 5.26) |
| Poly app users* outness | 2.59 (1.60 – 4.21) |
| Low cost app users* outness | 1.03 (0.86 – 1.23) |
|  |  |
| Gay app users* social support | 6.70 (0.77 – 58.7) |
| Poly app users* social support | 3.32 (0.88 – 12.5) |
| Low cost app users* social support | 0.92 (0.58 – 1.44) |
| Outness* social support | 1.00 (0.97 – 1.03) |
|  |  |
| Gay app users*outness* social support | 0.79 (0.65 – 0.98) |
| Poly app users*outness * social support | 0.67 (0.53 – 0.83) |
| Low cost app users*outness * social support | 1.00 (0.94 – 1.05) |
| *Significant at <0.05  **Significant at <0.001  Model 2a to 2b: adjusted for education, employment status, income, sold sex, steady partnership, any STI diagnosis, CAS in past 3months  R^2^ Nagelkerke (total variance explained) for models 1a to 2c: 0.027, 0.082, 0.115, 0.299, 0.318, 0.344 | |

| Supplementary Table 7: Models to examine the moderating effects of online sex-seeking patterns on last condomless anal sex without PrEP or ART with split samples at 70-90% | | | |
| --- | --- | --- | --- |
|  | **aPR (95% CI)** | **aPR (95% CI)** | **aPR (95% CI)** |
|  | Split sample at 90% | Split sample at 80% | Split sample at 70% |
| **Main effects** | N=905 | N=804 | N=704 |
| Negligible app users | Ref | Ref | Ref |
| Gay app users | 0.00 (0.00 – 5.08) | 0.01 (0.00 – 14.1) | 0.00 (0.00 – 1.58) |
| Poly app users | 0.05 (0.00 – 1.26) | 0.05 (0.00 – 4.74) | 0.16 (0.00 – 6.53) |
| Low cost app users | 1.03 (0.21 – 5.05) | 1.54 (0.30 – 7.97) | 1.07 (0.20 – 5.84) |
| Outness | 0.95 (0.85 – 1.06) | 0.96 (0.84 – 1.09) | 0.98 (0.88 – 1.10) |
| Social support | 0.84 (0.64 – 1.10) | 0.85 (0.60 – 1.20) | 0.83 (0.61 – 1.13) |
| **Main effects and 3-way interaction** |  |  |  |
| Gay app users* outness | 2.08 (0.95 – 4.58) | 1.95 (0.92 – 4.13) | 5.6 (1.42 – 22.0)* |
| Poly app users* outness | 2.65 (1.65 – 4.26)** | 2.66 (1.51 – 4.68) | 2.11 (1.22 – 3.65)** |
| Low cost app users* outness | 1.02 (0.83 – 1.26) | 1.05 (0.86 – 1.28) | 1.10 (0.91 – 1.33) |
|  |  |  |  |
| Gay app users* social support | 5.33 (0.73 – 38.9) | 3.25 (0.45 – 23.4) | 19.4 (0.69 – 543) |
| Poly app users* social support | 3.51 (0.98 – 12.6) | 3.39 (0.68 – 16.9) | 2.09 (0.48 – 9.15) |
| Low cost app users* social support | 0.93 (0.56 – 1.57) | 0.9 (0.52 – 1.56) | 1.02 (0.58 – 1.81) |
| Outness* social support | 1.00 (0.97 – 1.04) | 1.01 (0.97 – 1.04) | 1.00 (0.97 – 1.04) |
|  |  |  |  |
| Gay app users*outness* social support | 0.81 (0.67 – 0.99)* | 0.83 (0.67 – 1.02) | 0.63 (0.43 – 0.92)** |
| Poly app users*outness * social support | 0.66 (0.53 – 0.82)** | 0.66 (0.52 – 0.84)** | 0.73 (0.57 – 0.94)** |
| Low cost app users*outness * social support | 1.00 (0.94 – 1.06) | 0.99 (0.93 – 1.05) | 0.97 (0.91 – 1.03) |
| *Significant at <0.05  **Significant at <0.001  All models adjusted for education, employment status, income, sold sex, steady partnership, any STI diagnosis, CAS in past 3months | | | |
